# Spectroscopic Fingerprinting of Coordination‐driven Spin States in Metal‐organic Architectures

**DOI:** 10.1002/chem.202502828

**Published:** 2025-12-12

**Authors:** Yan Yan Grisan Qiu, Silvia Carlotto, Simone Mearini, Daniel Baranowski, Iulia Cojocariu, Matteo Jugovac, Giovanni Zamborlini, Pierluigi Gargiani, Manuel Valvidares, Vitaliy Feyer, Claus Michael Schneider

**Affiliations:** ^1^ Peter Grünberg Institute (PGI‐6) Jülich Research Center Jülich Germany; ^2^ Department of Chemistry University of Padova Padova Italy; ^3^ Institute of Condensed Matter Chemistry and Technologies for Energy (ICMATE) CNR C/O Department of Chemistry University of Padova Padova Italy; ^4^ Dipartimento Di Fisica Università degli Studi di Trieste Trieste Italy; ^5^ Institute of Physics NAWI Graz University of Graz Graz Austria; ^6^ Department of Physics TU Dortmund University Dortmund Germany; ^7^ ALBA Synchrotron Light Source Barcelona Spain; ^8^ Faculty of Physics and Center for Nanointegration Duisburg‐Essen (CENIDE) University of Duisburg‐Essen Duisburg Germany; ^9^ Department of Physics and Astronomy UC Davis Davis USA

**Keywords:** coordination geometry, low‐dimensional metal‐organic architecture, spin states, transition metal complex

## Abstract

Determining the local geometry of metal‐organic architecture on substrates is challenging, as substrate interactions can alter the metal coordination relative to the free‐standing structure. Here, combining density functional theory (DFT) and restricted open‐shell configuration interaction with singles (ROCIS) calculations on isolated cobalt‐7,7,8,8‐tetracyanoquinodimethane (Co‐TCNQ) complexes, together with X‐ray absorption spectroscopy (XAS) and X‐ray magnetic circular dichroism (XMCD), directly reveals the coordination motifs of Co centers in a 2D Co‐TCNQ framework on graphene. The calculated Co L_3,2_‐edges spectroscopic fingerprints for nearly planar (Co^2+^, S = 1/2) and distorted tetrahedral (Co^2+^, S = 3/2) structures exhibit distinct features, allowing unambiguous assignment of spin and oxidation states of the metal centers, as well as confirmation of the local geometry. Comparison with experimental spectra confirms that the high‐spin tetrahedral geometry is realized in the supported framework, demonstrating how spectroscopic fingerprints can directly link coordination geometry to spin and oxidation states in low‐dimensional metal‐organic systems.

## Graphical abstract

Coordination geometry controls spin states in Co‐TCNQ frameworks. Combined theory and XAS confirm the high‐spin Co^2+^ on graphene and establish a direct approach for spin‐state assignment in metal‐organic systems.

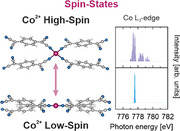

## 1 Introduction

The coordination environment of transition‐metal (TM) centers plays a decisive role in defining the electronic and magnetic properties of molecular complexes and low‐dimensional metal‐organic architectures [1, 2, 3, 4, 5, 6]. Subtle variations in the ligand arrangement provide a powerful means of tuning these properties [7, 8, 9, 10], as even small changes in coordination geometry can alter the metal oxidation state [11, 12, 13], electronic configuration [14, 15], and magnetic response [16, 17, 18, 19].

In molecular complexes, structural distortions frequently mediate redox processes at the metal site, stabilizing distinct oxidation and spin states [1, 6, 20, 21, 22]. A prominent case is ruthenium tetraphenylporphyrin (RuTPP) on Ag(111), where the saddle‐shaped conformation enables axial CO binding in contrast to the planar form, directly coupling the conformation to the electronic configuration [23].

Comparable effects have been recently extended to 2D metal‐organic frameworks (2D MOF), where substrate interaction determines whether structural flexibility is preserved or suppressed. For instance, cobalt porphyrin frameworks assembled on graphene/Ir(111) exhibit multiple oxidation states with CO reactivity [24], while Fe‐TCNQ frameworks on weakly interacting graphene/Ir(111) display greater conformation variability compared with the more rigid counterparts on Au(111) [1, 25]. These studies highlight that small distortions in coordination geometry can critically influence electronic and magnetic states. Establishing a clear correlation between local geometry and spin state, however, requires a combined experimental and theoretical analysis. Notably, weakly interacting substrates such as graphene/Ir(111) allow a wider range of conformations, as well as oxidation and spin states, since they impose fewer constraints on the arrangement of the supported species.

X‐ray absorption spectroscopy (XAS) at the TM L_3,2_‐edges provides element‐specific sensitivity to oxidation state, electronic configuration, and ligand field symmetry [26, 27, 28, 29, 30, 31]. When complemented by X‐ray magnetic circular dichroism (XMCD), quantitative information on spin states and magnetic moments can be obtained [16, 17, 18, 19, 32]. On the theoretical side, density functional theory (DFT) combined with restricted open‐shell configuration interaction with singles (ROCIS) provides accurate spectral simulations that capture fingerprints of oxidation and spin states [33]. This methodology has been successfully applied to the TM complexes of V [34], Mn [35], Fe [11, 36], Co [37, 38, 39, 40], and metal‐TCNQ derivatives [26], establishing a robust framework for interpreting experimental spectra. Despite this progress, applications to extended, substrate‐supported 2D MOFs remain largely unexplored.

Here, we address this gap by combining theory and spectroscopy to investigate the cobalt‐TCNQ system supported on graphene. DFT/ROCIS calculations are used to establish the spectroscopic fingerprints of distinct coordination motifs on isolated Co‐TCNQ complexes. These theoretical benchmarks then guide the interpretation of experimental spectra of the extended framework. By disentangling the intrinsic electronic effects of coordination geometry from substrate interactions, we directly link the spin and oxidation state of the Co centers to the local geometry.

## 2 Results and Discussion

Coordination environments in metal‐organic complexes and frameworks can be finely tuned through the use of flexible ligands such as TCNQ, whose adaptability accommodates geometrical distortions that significantly impact metal‐ligand interactions [1, 22]. Such distortions are often essential for redox activity, enabling the stabilization of distinct oxidation and spin states [23, 41].

**FIGURE 1:**
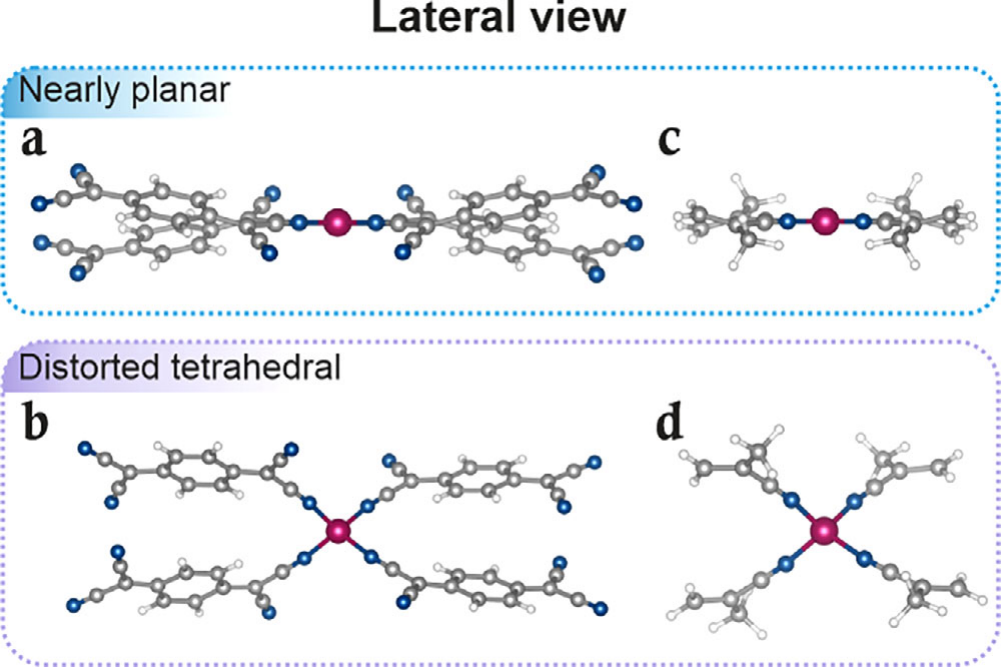
(left) Co‐TCNQ structures with four TCNQ ligands for (a) LS nearly planar and (b) HS distorted tetrahedral coordinative environments (lateral view). (right) Small cluster models for (c) LS nearly planar and (d) HS distorted tetrahedral structures were used to simulate the experimental Co L_3,2_‐edges XAS spectra. Grey, white, blue, and magenta spheres correspond to C, H, N, and Co atoms, respectively.

To establish reliable structural models with well‐defined electronic properties, we first examined isolated Co‐TCNQ coordination units. Structural optimizations reveal two stable motifs: a nearly planar environment and a distorted tetrahedral configuration (Figures 1a, b and Figure S1). All LS initial geometries converged to the planar structure, while the HS starting geometries consistently relaxed into the distorted tetrahedral form.

In the nearly planar geometry, the Co‐N bond length is 1.895 Å, and the average N‐Co‐N bond angle is close to 90°, with all four nitrogen atoms lying nearly coplanar with the Co center. The term “nearly planar” reflects a ∼30° tilt of the TCNQ ligands relative to the plane defined by the Co and its four coordinating N atoms. The distorted tetrahedral configuration exhibits longer Co‐N bonds (1.977 Å) and N‐Co‐N angles ranging from 102.1° to 115.6° (average 109.6°), while retaining a similar ∼30° ligand tilt. Energetically, the HS tetrahedral motif is favored by ∼0.5 eV.

Given the high sensitivity of the Co L_3,2_‐edges XAS to oxidation and spin states, the optimized structural motifs were used as the basis for cluster multiplet calculations. The simulated spectra exhibit distinct spectroscopic fingerprints for the low‐spin (LS) and high‐spin (HS) configurations, reflecting their different 3d electronic occupations. These calculated features provide an unambiguous criterion for distinguishing between spin states in the experiment.

Before discussing the Co L_3,2_‐edges, we first consider the XAS spectra measured across the N K‐edge, which provide direct information on ligand coordination and charge transfer between the Co and TCNQ ligands during MOF formation. Figure S2 presents N K‐edge spectra measured with p‐ and s‐polarized light for both self‐assembled TCNQ molecules and the Co‐TCNQ MOF on graphene/Ir(111).

For the pristine TCNQ layer, the p‐polarized spectrum shows three π*‐type resonances at 396.9 eV, 398.9 eV, and 399.9 eV. The first and third resonances are associated with orbitals localized on the quinoid ring, whereas the intermediate feature arises from π*‐orbitals of the cyano groups (CN) [42, 43]. The spectrum measured with s‐polarized light displays a single intense resonance at 398.8 eV, corresponding to in‐plane π*‐type orbitals localized on the cyano groups [22]. The pronounced linear dichroism indicates a preferential planar orientation of the TCNQ molecules on the substrate.

Metal coordination induces a marked transformation of the N K‐edge spectral line shape. The π*‐type orbitals localized on the quinoid ring (at 396.9 eV and 399.9 eV) become partially occupied due to charge transfer from the Co metal to the TCNQ ligand, while the cyano‐group resonances evolve into two distinct features at 398.4 eV and 399.6 eV in s‐ and p‐polarized spectra, respectively [8, 42, 43]. These peaks correspond to orthogonal π*‐symmetry orbitals of the CN groups that are degenerate in isolated TCNQ molecules [42, 43]. The observed splitting reflects substantial charge redistribution and hybridization between Co 3d and cyano‐group π* orbitals, confirming extended MOF formation and providing direct spectroscopic evidence of metal‐ligand coordination [44, 45].

Both π*‐symmetry orbitals remain orthogonal to the CN bond axis, with the low‐energy resonance oriented parallel to the axis plane and the high‐energy resonance perpendicular to it [8, 42, 43]. Notably, the low‐energy resonance exhibits significant intensity in both polarization geometries. The XAS intensity ratio (Figure S2) in p‐ and s‐polarization ($I_{p}/I_{s}$) follows the relation $I_{p}/I_{s}$ ∝ tan^2^γ, where γ represents the tilt angle between the CN bond axis and the substrate surface [46]. Quantitative analysis of the N K‐edge XAS dichroism yields an $I_{p}/I_{s}$ ratio of approximately 0.32 for this resonance, corresponding to a CN bond tilt of about 29° from the substrate plane, thus confirming the substantial out‐of‐plane distortion.

Previous studies combining scanning tunneling microscopy (STM) and low‐energy electron diffraction (LEED) on TM‐TCNQ (M‐TCNQ) 2D MOFs on graphene (M = Ni, Fe, or Mn) have revealed distinct diffraction patterns and established a characteristic coordination motif in which each metal center is coordinated by four nitrogen atoms from four different TCNQ ligands [1, 47]. Our LEED measurements (Figure S3) show clear modifications to the diffraction pattern of the pristine TCNQ layer upon cobalt deposition. The resulting LEED pattern of the Co‐TCNQ framework closely resembles that reported for the analogous Ni‐TCNQ system on graphene [47]. Based on this structural similarity, we conclude that the Co‐TCNQ network formed under our preparation conditions adopts a comparable fourfold coordination geometry, corresponding to a 1:1 stoichiometry ratio (Co(TCNQ)).

Although the framework is periodic, the local Co environment can be well represented by the planar and distorted motifs described above. Due to the localized nature of the core‐level excitations and the weak interaction of the 2D MOF with the graphene substrate [26], the small‐cluster models provide reliable spectroscopic fingerprints for interpreting the data from the extended framework (Figures 1c,d).

Comparison of the calculated spectra with experimental Co L_3,2_‐edges XAS allows us to identify the actual spin state. While previous STM studies have reported the coexistence of planar and distorted coordination environments in related Fe‐TCNQ MOFs on graphene/Ir(111) [1, 47], STM alone cannot resolve the oxidation or spin state of the metal centers. Our combined DFT/ROCIS with XAS approach overcomes this limitation by directly linking local geometry to unambiguous spectral fingerprints.

**FIGURE 2:**
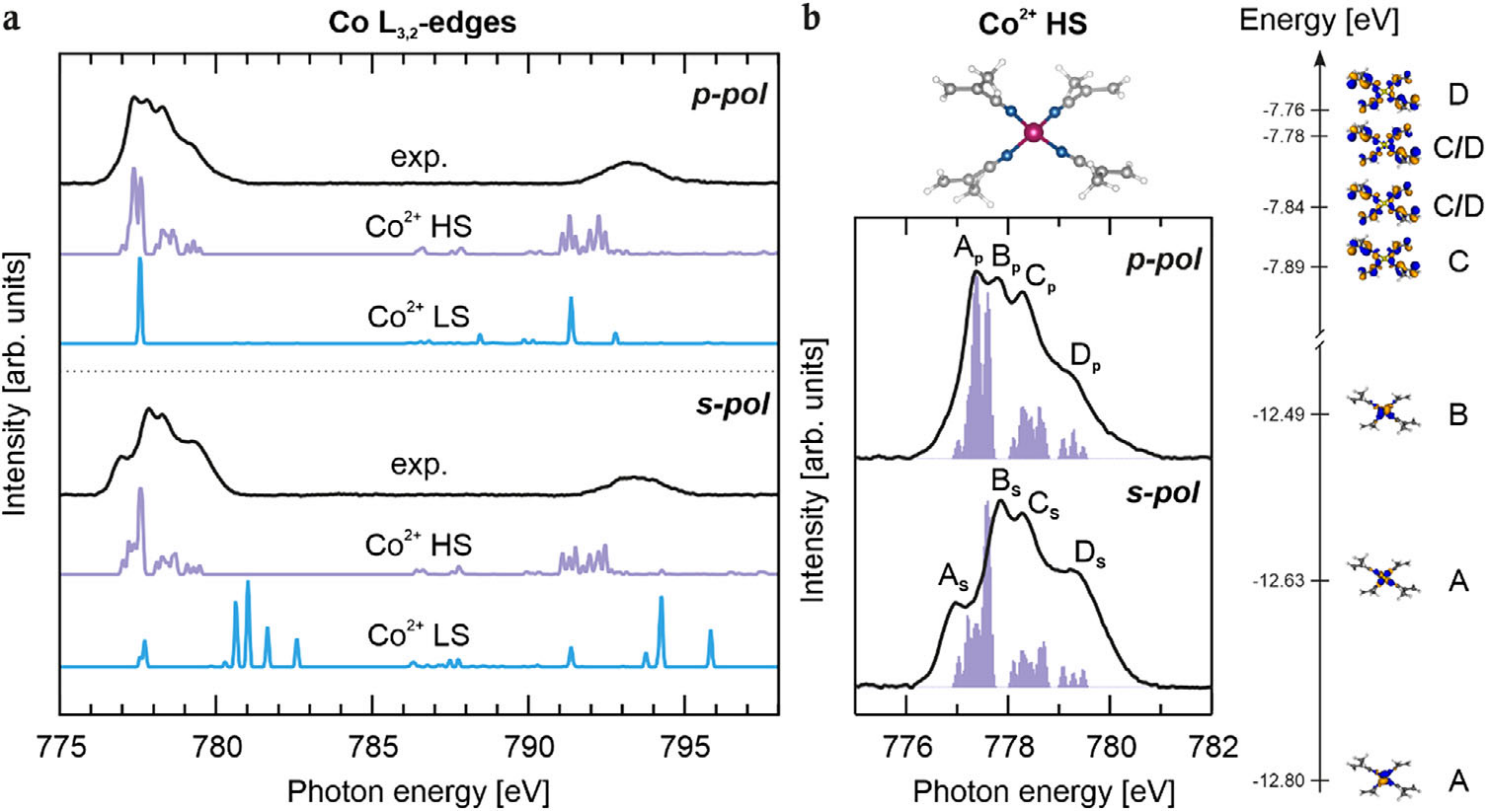
(a) Comparison of experimental (black) and simulated Co^2+^ HS (purple) and Co^2+^ LS (cyan) Co L_3,2_‐edges XAS spectra. The top and bottom panels correspond to p‐ and s‐polarization, respectively. The simulated spectra have been shifted by 16.91 eV to align (p‐polarization) the maxima of the simulated L_3_ lines with the corresponding experimental ones. A Gaussian broadening of 0.1 eV has been applied to all simulated spectra. (b) Comparison of the measured Co^2+^ L_3_ absorption lines (black) with the corresponding simulated bars (purple) obtained by assuming a distorted tetrahedral structure and an HS configuration (inset above the spectra). The p‐ and s‐polarizations are reported in the top and bottom panels, respectively. See the text for a detailed analysis of the main contributions to the bars. 3D plots displayed isosurfaces corresponding to ±0.03 e^1/2^ Å^3/2^ (see Figure S4 for an enlarged view).

A detailed comparison reveals that the simulated spectra for the LS state (S = 1/2) fail to reproduce the complex multiplet features observed experimentally (Figure 2a). Under p‐polarization, the LS simulation shows only a single broad peak at the L_3_‐edge, and although multiple peaks appear in s‐polarization, their positions do not match the experiment. Furthermore, the broad experimental band at the L_2_‐edge, indicative of multiple transitions, is absent in the LS simulation. In contrast, the simulated spectra for the HS state (S = 3/2) exhibit stronger agreement with the experimental observations. The HS simulations accurately reproduce the multi‐peak structures observed under both polarization conditions. Under p‐polarization at the L_3_‐edge, two low‐energy peaks with a higher‐energy shoulder are clearly resolved, while s‐polarization yields a central intense peak flanked by two shoulders, in good agreement with the experiment (Figures 2a,b). Minor deviations, such as the underestimated L_3_‐L_2_ energy splitting and the reduced intensity of the L_3_ satellite peak (Figure 2a), originate from known limitations of the DFT/ROCIS method, including the underestimation of 2p spin‐orbit coupling and incomplete treatment of the multiplet effect [33].

The strong agreement between HS simulations and experiment unambiguously confirms that the Co^2+^ centers in the Co‐TCNQ MOF adopt a high‐spin configuration with three unpaired electrons. This electronic state is structurally correlated with a distorted tetrahedral coordination environment, demonstrating that the stabilization of the HS state in the 2D MOF originates from a combination of local geometry and metal‐ligand hybridization.

A closer inspection of the L_3_‐edge region (775‐782 eV) provides further microscopic insight into the electronic structure (Figure 2b). Peaks $A_{p}$ and $A_{s}$ (776.6‐777.6 eV) originate from transitions into the two singly occupied molecular orbitals (SOMOs), the $3d_{xz}$ and $3d_{yz}$. The most intense resonance in s‐polarization, $B_{s}$, and the $B_{p}$ peak (777.6‐778.1 eV) include transitions into the highest energy SOMO, the $3d_{xy}$ orbital. The distortion induced by TCNQ ligands lifts the degeneracy of these orbitals, leading to the observed multiple peaks in both the experimental and simulated spectra. The calculated energy separation between the $3d_{xz}$ orbital (identified as the lowest energy SOMO) and the $3d_{yz}$ and $3d_{xy}$ orbitals are 0.17 eV and 0.31 eV, respectively. The higher energy features $C_{s}$ and $C_{p}$ (778.1‐778.8 eV), and $D_{s}$ (778.8 eV), arise predominantly from ΔS=−1 transitions into virtual molecular orbitals (VMOs) of metal‐to‐ligand‐charge transfer (MLCT) character.

In summary, the N K‐edge analysis confirms 2D MOF formation through charge transfer and hybridization, while the Co L_3,2_‐edges analysis establishes the high‐spin Co^2+^ state. The low‐energy L_3_‐edge features (A and B) reflect localized excitation into Co 3d orbitals, whereas the higher‐energy resonances (C and D) involve MLCT processes.

**FIGURE 3:**
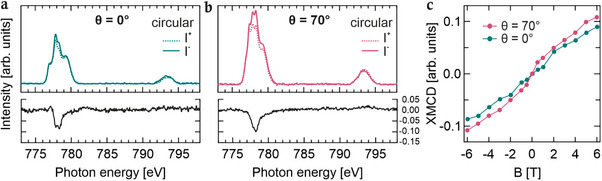
XAS spectra at the Co L_3,2_‐edges of left‐ ($I^{-}$, solid line) and right‐ ($I^{+}$, dashed line) hand circularly polarized light (B = 6 T) at (a) normal incidence (θ = 0°, green) and (b) grazing incidence (θ = 70°, pink), and resulting XMCD signal (normalized by the corresponding L_3_‐integrated XAS intensity, black). A spline background has been subtracted. (c) Experimental magnetization curves intensity integrated over the L_3_‐edge (normalized by the corresponding L_3_‐integrated XAS intensity) vs. the magnetic field B at normal (θ = 0°, green) and grazing (θ = 70°, pink) incidence geometries and temperature of 5 K.

Given that the coordination environment of the Co center strongly dictates its spin configuration [48, 49, 50], we further investigated the magnetic properties of the Co^2+^ ions by XMCD combined with sum rule analysis. The measurements were carried out at 5 K under an applied magnetic field of up to 6 T across the Co L_3,2_‐edges (Figures 3a,b). The spectra were collected in two experimental geometries, corresponding to photon incidence angles of θ = 0° (normal incidence) and θ = 70° (grazing incidence) relative to the sample surface normal.

The XMCD spectra exhibit a pronounced angular dependence. At normal incidence, a weak shoulder at 777.1 eV together with a multiplet structure is observed, whereas at grazing incidence, this complexity collapses into a single dominant peak. Such geometry‐dependent spectral fingerprints are characteristic of HS Co^2+^ ions, reflecting anisotropic orbital contributions to the magnetic response. The two leading low‐energy resonances at the L_3_‐edge show distinct intensity variations between geometries, highlighting the role of the $3d_{xz}$, $3d_{yz}$, and $3d_{xy}$ orbitals in shaping the anisotropy.

To quantify the orbital and the effective spin magnetic moments, a sum rule analysis was applied to the XAS and XMCD spectra acquired at both incidence geometries (Table 1 and Figure S5). The extracted values correspond to the magnetic moments projected onto the X‐ray incidence direction, assuming a nominal d‐hole count of three for high‐spin Co^2+^. Uncertainties were estimated by independently varying the integration limits for both XMCD and XAS spectra. The oxidation state of cobalt was independently verified from the Co $L_{3}/\left(L_{3}+L_{2}\right)$ branching ratio, resulting in the range of 0.78‐0.80. This value is significantly higher than the atomic value of 2/3 [51], thereby confirming the high‐spin Co^2+^ (3d^7^, $n_{h}$ = 3) configuration.

**TABLE 1:** Sum‐rule derived magnetic moments from the XMCD spectra measured at T = 5 K, B = 6 T of Co‐TCNQ MOF/graphene/Ir(111) system for θ = 0° (normal incidence) and θ = 70° (grazing incidence). Magnetic moment values are given in $\mu_{B}$/atom.

Co MOF	$\theta =0^{\circ }$	$\theta =70^{\circ }$
$2\left\langle S_{z}\right\rangle +7\left\langle T_{z}\right\rangle$	(0.30±0.01)	(0.62±0.09)
$\left\langle L_{z}\right\rangle$	(0.05±0.01)	(0.11±0.03)

The effective spin magnetic moment includes a magnetic dipole term, which can lead to deviations between the effective spin moment and the spin moment [18, 32]. Consequently, the values summarized in Table 1 should be regarded as lower limits, since full magnetic saturation was not reached under the present conditions [32]. The experimentally derived moments are smaller than the expected theoretical values for Co^2+^ ions (S = 3/2), as reported in Ref [52]. The XMCD‐derived magnetization curves (Figure 3c) recorded at the maximum L_3_‐edge XMCD intensity exhibit a nearly linear field dependence up to 6 T, remaining far below the expected saturation limit for an S = 3/2 system, as described by the Brillouin function [52]. This deviation indicates that, under the investigated conditions, the system behaves as a purely paramagnetic material with no evidence of magnetic saturation, thereby precluding access to the real magnetic moments, which can only be determined under full saturation. This trend, together with the calculated magnetic moments, is consistent with previous observations in other low‐dimensional Co‐based MOFs [48, 49, 53], where the magnetization curves similarly remain far from saturation even at 6 T, thus leading to comparatively low magnetic moments extrapolated from the XMCD measurements.

## 3 Conclusion

In summary, Co L_3,2_‐edges XAS and XMCD, supported by DFT/ROCIS calculations, provide a spectroscopic route to determine the local geometry, spin state, and oxidation state of Co centers in a 2D Co‐TCNQ framework on graphene. The spectra establish that the Co^2+^ ions adopt a high‐spin state stabilized by a distorted tetrahedral coordination environment, with XMCD confirming geometry‐dependent magnetic anisotropy and paramagnetic behavior. More broadly, this combined approach resolves both coordination geometry and electronic configuration in substrate‐supported metal‐organic systems.

### 3.1 Experimental Sections/Methods

### 3.2 Sample Preparation

The Ir(111) single crystal was cleaned by argon ion (Ar⁺) sputtering at room temperature, followed by annealing to 1500 K. The sputtering was conducted at an energy of 1.5‐2.0 keV.

Graphene was synthesized via chemical vapor deposition (CVD) using ethylene (C_2_H_4_) gas. Ethylene was initially introduced into the preparation chamber at a pressure of 5 × 10^−8^ mbar for 2 min, after which the pressure was increased to 1 × 10^−6^ mbar for 5 min. The substrate temperature was maintained at 1400°C during the growth process. The quality of graphene was checked by LEED.

The Co‐TCNQ framework was prepared following the growth protocol established by Jakub et al. [47] for M‐TCNQ (M = Ni, Fe, or Mn) 2D MOFs on graphene/Ir(111). TCNQ molecules were thermally evaporated from a Knudsen‐type evaporator at 410 K onto the graphene substrate held at 300 K. Subsequently, cobalt was evaporated from an e‐beam evaporator at a nominal coverage of 0.04 ML (ion fluxes ∼10 nA), followed by annealing to 475 K. This annealing step promotes the formation of the extended Co‐TCNQ network and facilitates the desorption of noncoordinated TCNQ species, thereby driving the system toward a 1:1 metal‐to‐ligand stoichiometry. Previous STM studies have demonstrated that M‐TCNQ frameworks remain stable up to ∼530 K [47], confirming that the applied annealing temperature does not disturb the coordinated phase. Moreover, the multiplet structure and pronounced polarization dependence of Co L‐edge spectra in this work are markedly different from those of metallic or cluster‐like Co species [54], confirming that Co clustering does not occur in appreciable amounts under these conditions.

### 3.3 Experimental Methods

XAS and XMCD measurements were conducted at the BOREAS beamline of the ALBA synchrotron [55]. Both XAS and XMCD spectra were collected in total electron yield mode at the Co L_3,2_‐edges. The signal was normalized by the total electron yield measured on a diamond membrane placed between the last focusing mirror and the sample. In the XAS experiment, both transverse electric (s‐polarization) and transverse magnetic (almost p‐polarization) polarization experiments were performed by changing from linear vertical to linear horizontal polarizations. The XAS was performed with the sample kept at 300 K in order to suppress substrate‐related oscillations originating from the Ir N‐edge extended X‐ray absorption fine structure. At lower temperatures, the reduced Debye‐Waller damping enhances the amplitude of these oscillations [56], which can interfere with the detection of weak molecular absorption features. Performing the measurements at 300 K minimizes these contributions and yields smoother background conditions for quantitative analysis.

For the XMCD signals, obtained by subtracting the XAS spectra collected with positive helicity ($I^{+}$) from those with negative helicity ($I^{-})\;$ at the Co L_3,2_‐edges, a magnetic field of B = 6 T and a temperature of approximately 5 K were used. The magnetic field was applied parallel to the X‐ray beam, defining the photon propagation direction as the quantization axis. Consequently, the extracted values correspond to the magnetic moments projected onto the X‐ray incidence direction. To prevent beam‐induced damage, the sample was continuously moved to expose fresh, nonilluminated areas.

### 3.4 Computational Details

Geometry optimizations: All quantum mechanical calculations were carried out by using the ORCA program (version 5.0.0) [57]. All structures were optimized using the hybrid B3LYP functional [58] in connection with the def2‐TZVP(‐f) basis set [59], where def2‐TZVP is a valence triple‐zeta basis set with “new” polarization functions. Dispersion corrections were included by adopting Grimme's DFT‐D3 approach with D3BJ, an atom‐pairwise dispersion correction to the DFT energy with Becke‐Johnson damping [60]. In particular, Co‐TCNQ structures were modeled by considering Co^2+^ centers coordinated by four TCNQ ligands and evaluated for both low‐spin and high‐spin states, maintaining a +2 overall charge. In the absence of crystallographic data or a periodic structural model for the Co‐TCNQ, our approach is based on a recent gas‐phase modeling of TM‐based TCNQ MOFs. Jakub et al. [1] identified several accessible coordination motifs for Fe in a Fe‐TCNQ MOF, including planar, quasi‐tetrahedral twisted, and tilted geometries, which guided our exploration. Additionally, a displaced planar geometry, where the Co centers lie slightly out of the ligand plane, was also considered in the structural search.

XAS spectra simulations: Simulation of the Co L_3,2_‐edges XAS spectra was performed using the DFT/ROCIS method as implemented in the ORCA program package [33], which incorporates the spin‐orbit coupling (SOC) effect in a molecular Russell‐Saunders scheme. ROCIS calculation used standard semi‐empirical parameters (c_1_ = 0.21, c_2_ = 0.49, and c_3_ = 0.29) [57], and scalar relativistic effects were treated using the zeroth‐order regular approximation (ZORA) [61]. The resolution‐of‐identity approximation [62] with the def‐TZVP/J basis was applied throughout. Simulated spectra were energy‐shifted to align the most intense L_3_‐edge feature with the corresponding experimental resonance for direct comparison. The choice of functional and semi‐empirical ROCIS parameters can influence spectral features. To evaluate this sensitivity, additional calculations are performed using the PBE0 hybrid functional and an alternative set of ROCIS parameters (c_1_ = 0.18, c_2_ = 0.20, c_3_ = 0.40) [63] on both HS and LS states. Comparisons are reported and discussed in the Supporting Information.

## Supporting Information

The authors have cited additional references within the Supporting Information [64, 65].

## Conflicts of Interest

The authors declare no conflict of interest.

## Supporting information




**Supporting file 1**: chem70562‐sup‐0001‐SuppMat.docx
