# Supplementary material for: Spectroscopic Fingerprinting of Coordination‐driven Spin States in Metal‐organic Architectures
Source: Chemistry. 2025 Dec 12;32(3):e02828. doi: 10.1002/chem.202502828 (PMC12824828; doi:10.1002/chem.202502828)
Supplement: Supplementary file 1 — Supporting file 1: chem70562‐sup‐0001‐SuppMat.docx [file CHEM-32-e02828-s001.docx]

**Supporting Information:**

**Spectroscopic fingerprinting of coordination-driven spin states in metal-organic architectures**

*Yan Yan Grisan Qiu ^a^*, Silvia Carlotto ^b,c^*, Simone Mearini ^a^, Daniel Baranowski ^a,^*^d^*, Iulia Cojocariu ^e^, Matteo Jugovac ^e^, Giovanni Zamborlini ^f,g^, Pierluigi Gargiani ^h^, Manuel Valvidares ^h^, Vitaliy Feyer ^a,i^* and Claus Michael Schneider ^a,i,l^*

^a^ Peter Grünberg Institute (PGI-6), Forschungszentrum Jülich GmbH, 52425 Jülich, Germany

^b^ Department of Chemical Sciences, University of Padova, via F. Marzolo 1, 35131 Padova, Italy

^c^ Institute of Condensed Matter Chemistry and Technologies for Energy (ICMATE), National Research Council (CNR), c/o Department of Chemical Sciences, University of Padova, via F. Marzolo 1, 35131 Padova, Italy

^d^ Physical and Computational Sciences Directorate and Institute for Integrated Catalysis, Pacific Northwest National Laboratory, Richland, Washington 99354, USA (present address)

^e^ Physics Department, University of Trieste, 34127 Trieste, Italy

^f^ Institute of Physics, NAWI Graz, University of Graz, 8010 Graz, Austria

^g^ Department of Physics, TU Dortmund University, 44227 Dortmund, Germany

^h^ ALBA Synchrotron Light Source, 08290 Barcelona, Spain

^i^ Faculty of Physics and Center for Nanointegration Duisburg-Essen (CENIDE), University of Duisburg-Essen, 47048 Duisburg, Germany

^l^ Department of Physics and Astronomy, UC Davis, Davis CA 95616, USA

*Corresponding authors: y.grisan@fz-juelich.de, silvia.carlotto@unipd.it, v.feyer@fz-juelich.de

**S1. Co-TCNQ structures**


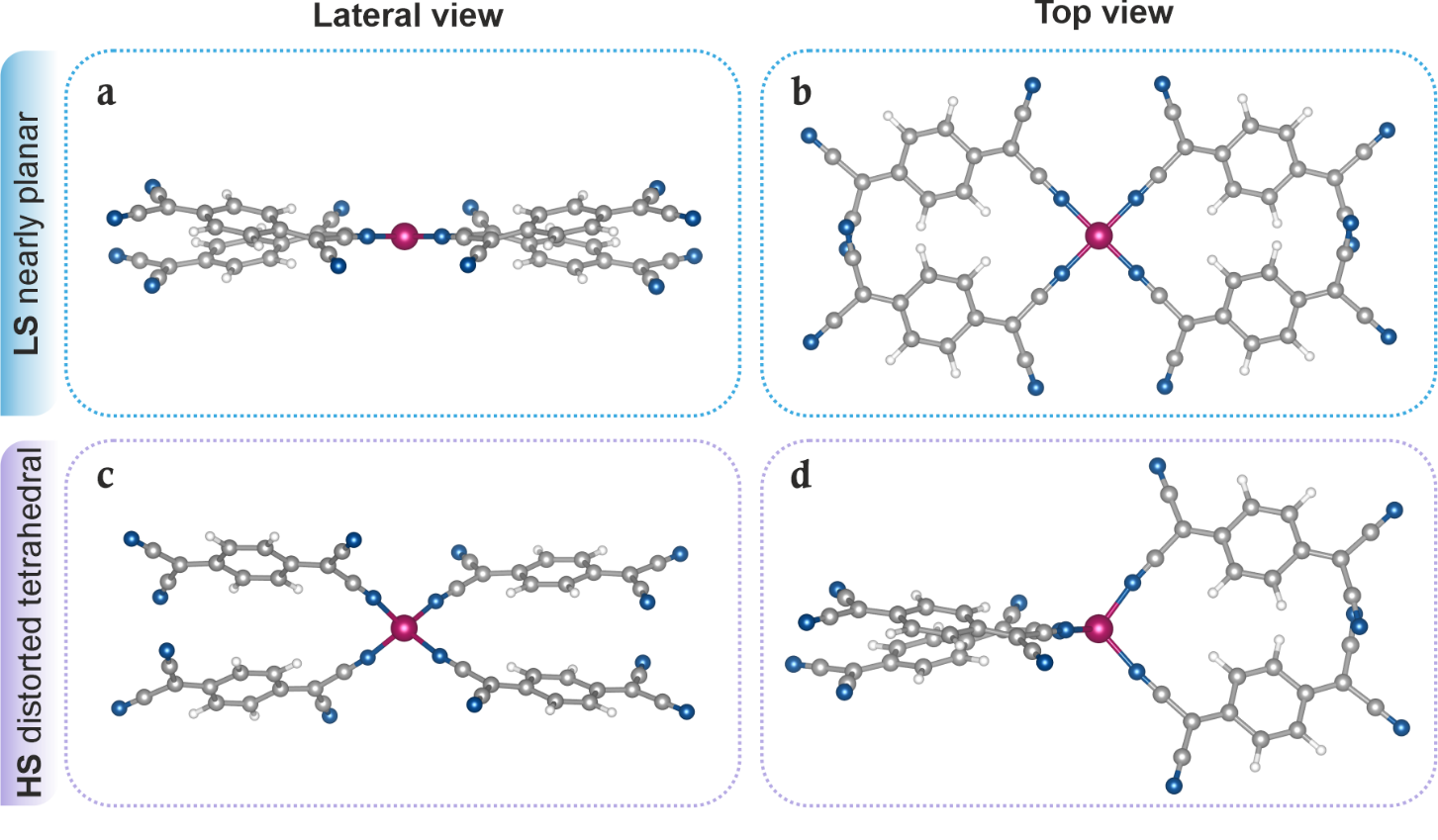


**Figure S1.** Lateral and top views for Co-TCNQ structures with four TCNQ ligands for (a,b) LS nearly planar and (c,d) HS distorted tetrahedral coordinative environments. Different views are reported to better characterize the structures. Grey, white, blue and magenta spheres correspond to C, H, N and Co atoms, respectively.

**S2. XAS spectra**


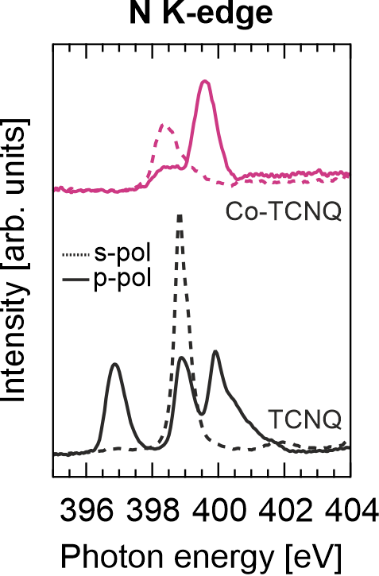


**Figure S2.** XAS spectra recorded across the N K-edge of the Co-TCNQ MOF/graphene/Ir(111) (magenta) and TCNQ/graphene/Ir(111) (black) systems, with s-polarized (s-pol, dashed line) and p-polarized (p-pol, solid line) light.

**S3.** **LEED patterns**


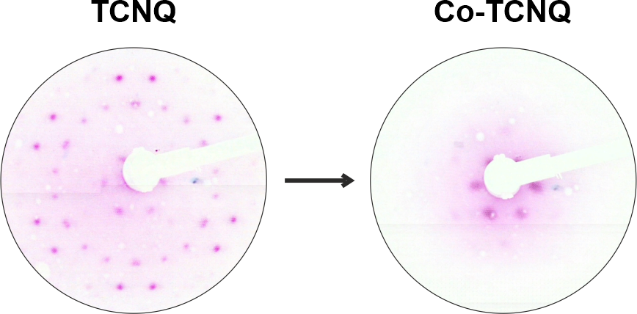


**Figure S3.** LEED patterns of TCNQ/graphene/Ir(111) and Co-TCNQ MOF/graphene/Ir(111) systems, acquired with an electron energy of 15 eV.

**S4. 3D plot displayed isosurfaces**


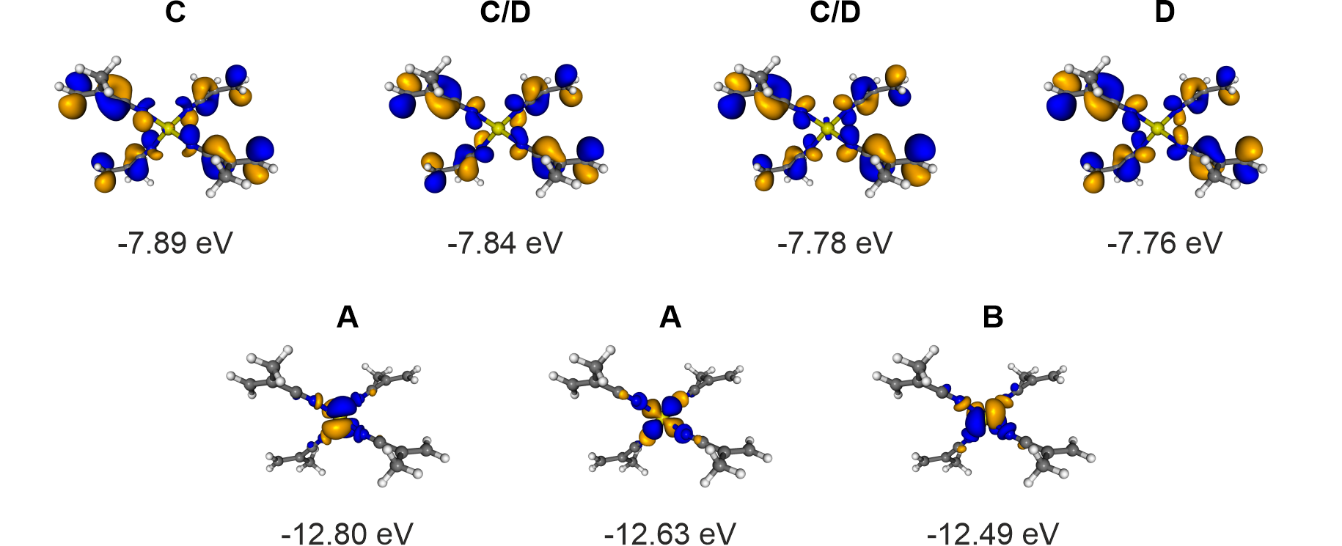


**Figure S4.** 3D plots displayed isosurfaces correspond to ±0.03 e^1/2^ Å^3/2^.

**S5. XMCD sum rule analysis**

The orbital and spin contributions to the total magnetic moment are expressed as:^[64,65]^

$$2\left\langle S_{z} \right\rangle+7\left\langle T_{z} \right\rangle=\frac{9\int_{L_{3}} (I^{+}-I^{-})dE - 6\int_{L_{3}+L_{2}} (I^{+}-I^{-})dE}{\int_{L_{3}+L_{2}} (I^{+}+I^{-}+I^{0})dE}n_{h}$$

$$\left\langle L_{z} \right\rangle=2\frac{\int_{L_{3}+L_{2}} (I^{+}-I^{-})dE}{\int_{L_{3}+L_{2}} (I^{+}+I^{-}+I^{0})dE}n_{h}$$

The expectation values of the spin $\left\langle S_{z} \right\rangle$, orbital $\left\langle L_{z} \right\rangle$and spin dipolar $\left\langle T_{z} \right\rangle$ moments of the Co 3d shell are obtained along the *z*-axis, defined by the incidence direction of the circularly polarized X-ray beam. The denominator corresponds to the integrated isotropic intensity: $I^{iso}=I^{+}+I^{-}+I^{0}$, where the term $I^{0}$ is related to the XAS component measured with the electric field vector parallel to the magnetization direction z. Since this value is generally not accessible in a single-magnet XMCD measuring setup, is approximated by the average $I^{0}= {(I^{+}+I^{-})}/2$, leading to the simplified expression: $I^{iso}=3/2 (I^{+}+I^{-})$. The magnetic moment values calculated along the applied magnetic field direction *z* at *T* = 5 K, *B* = 6 T, with the number of holes ($n_{h})$ set to three, are reported in Table 1 and Figure S5 for the two different geometries. The errors in the extracted magnetic moments were evaluated by varying the L_3_ and L_2_ integration limits over a few eV, to estimate the influence of involuntary offsets and noise in the XAS and XMCD baselines.


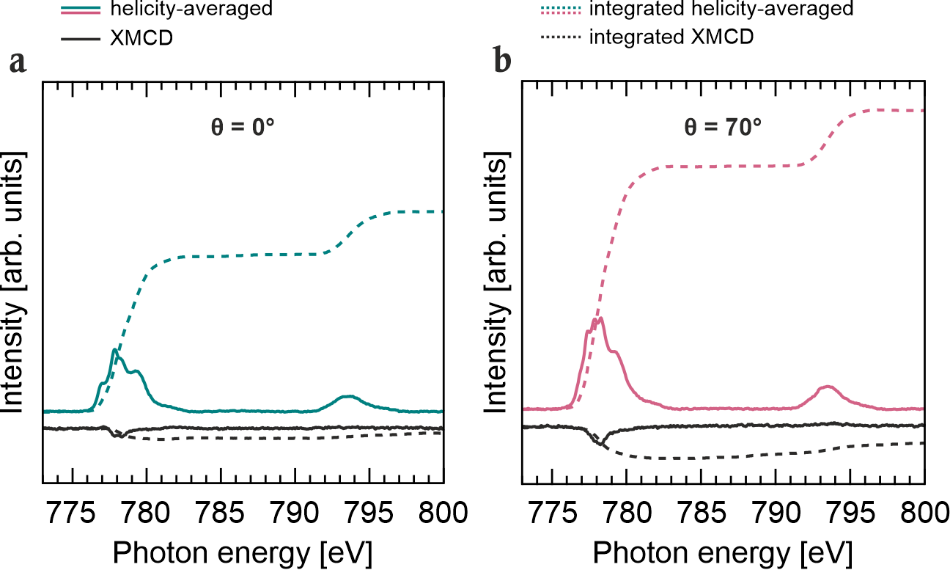


**Figure S5.** Integrated helicity-averaged and XMCD signals across the Co L_3,2_-edges absorption spectra at photon incidence angles of (a) θ = 0° (normal incidence) and (b) θ = 70° (grazing incidence) with respect to the surface normal obtained with B = 6 T and T = 5 K.

**S6. Method sensitivity tests**

**Influence of semi-empirical ROCIS parameters on the simulated Co L_3,2_-edges spectra**

For this Co complex and its two spin states, the choice of semi-empirical ROCIS parameters affects only slightly the simulated spectral features. Figures S6 and S7 compare results obtained with two different parameter sets for HS and LS states, respectively. For the Co^2+^ HS complex (Figure S6), the main differences between the two parameter sets are: a greater number of features in the L_2_ region for the set c_1_ = 0.21, c_2_ = 0.49, c_3_ = 0.29 for both polarizations, variations in the relative intensities between the L_3_ and L_2_ regions, and distinct changes in the L_3_ region, particularly in the lower-energy peak and the mid-energy range (around 778 eV). The L_3_-L_2_ energy separation is comparably underestimated in both parameter sets relative to the experimental spectra.


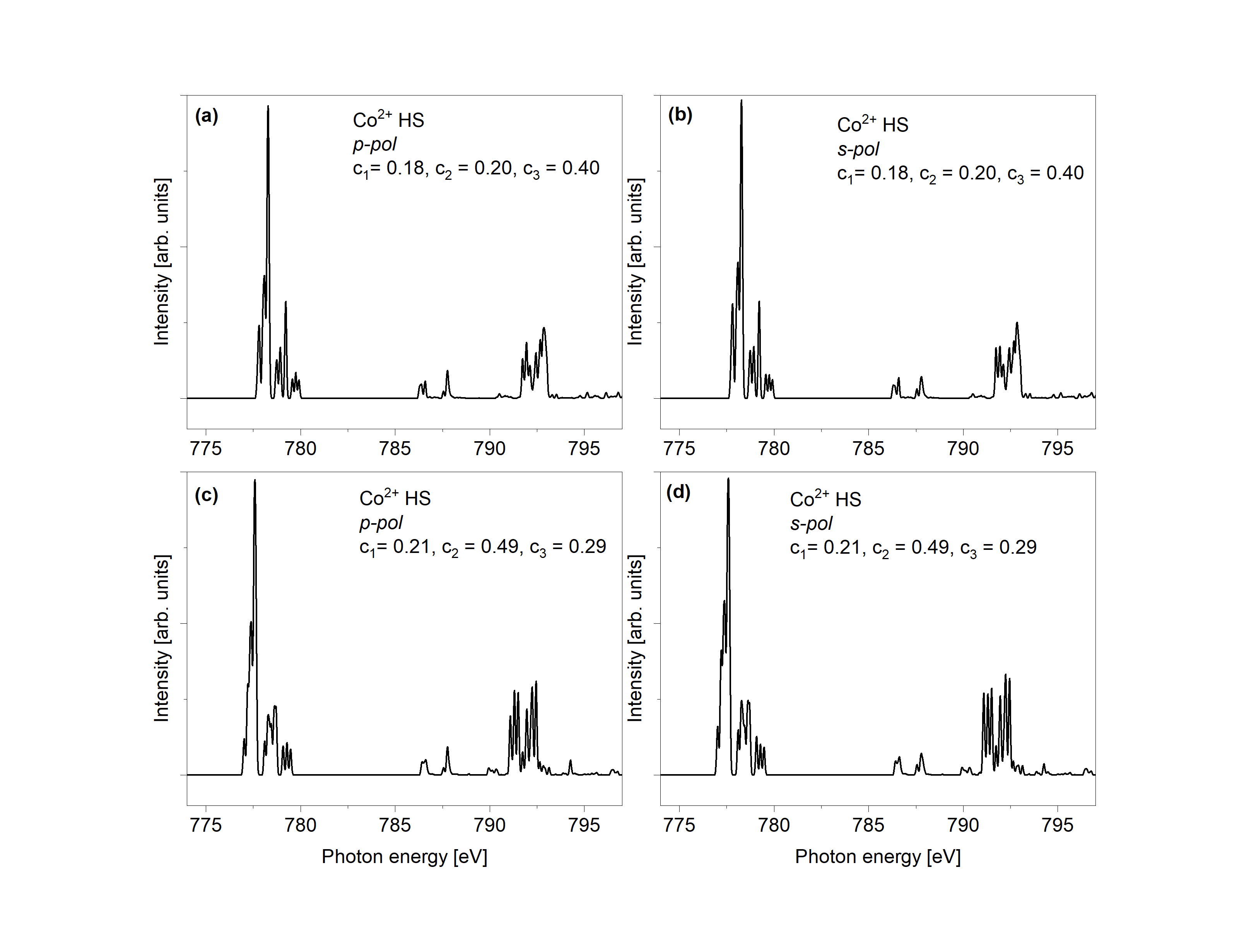


**Figure S6.** Comparison of Co^2+^ HS L_3,2_-edges XAS spectra calculated using two different sets of semi-empirical ROCIS parameters. Spectra obtained with (c_1_ = 0.18, c_2_ = 0.20, c_3_ = 0.40) are shown for (a) p-polarization and (b) s-polarization, while those calculated with (c_1_ = 0.21, c_2_ = 0.49, c_3_ = 0.29) are shown for (c) p-polarization and (d) s-polarization. All simulated spectra were shifted by +16.91 eV and convoluted with a Gaussian broadening of 0.1 eV.

Larger differences are present when comparing the two parameter sets for the Co^2+^ LS complex (Figure S7). Overall, the relative intensities between the L_2_ and L_3_ regions differ, with the L_2_ region showing higher intensity for the set c_1_ = 0.21, c_2_ = 0.49, c_3_ = 0.29. Moreover, this set exhibits an additional peak in the L_3_ region at 780.6 eV, which is absent in the alternative parameter set.

In conclusion, for both polarization directions and spin states, the simulated Co L_3,2_-edges XAS spectra demonstrate only marginal dependence on the specific choice of semiempirical ROCIS parameters. The overall spectral features, including the main peak positions, relative intensities, and L_3_-L_2_ energy separation, remain largely consistent across parameter sets. Noticeable variations arise only when employing a very narrow Gaussian broadening, which enhances subtle differences in the fine structure, particularly in the low-energy region of the L_3_-edge. However, as the spectral broadening increases, these differences become progressively less pronounced and are effectively masked within the overall band envelope. This observation suggests that the qualitative interpretation of the Co L_3,2_-edges spectra, such as the assignment of electronic transitions and oxidation/spin-state characterization, is robust with respect to small variations in the ROCIS parameterization. Therefore, while fine-tuning the parameters can slightly refine the spectral details, the general electronic structure trends and experimental agreement remain reliable across the tested configurations.


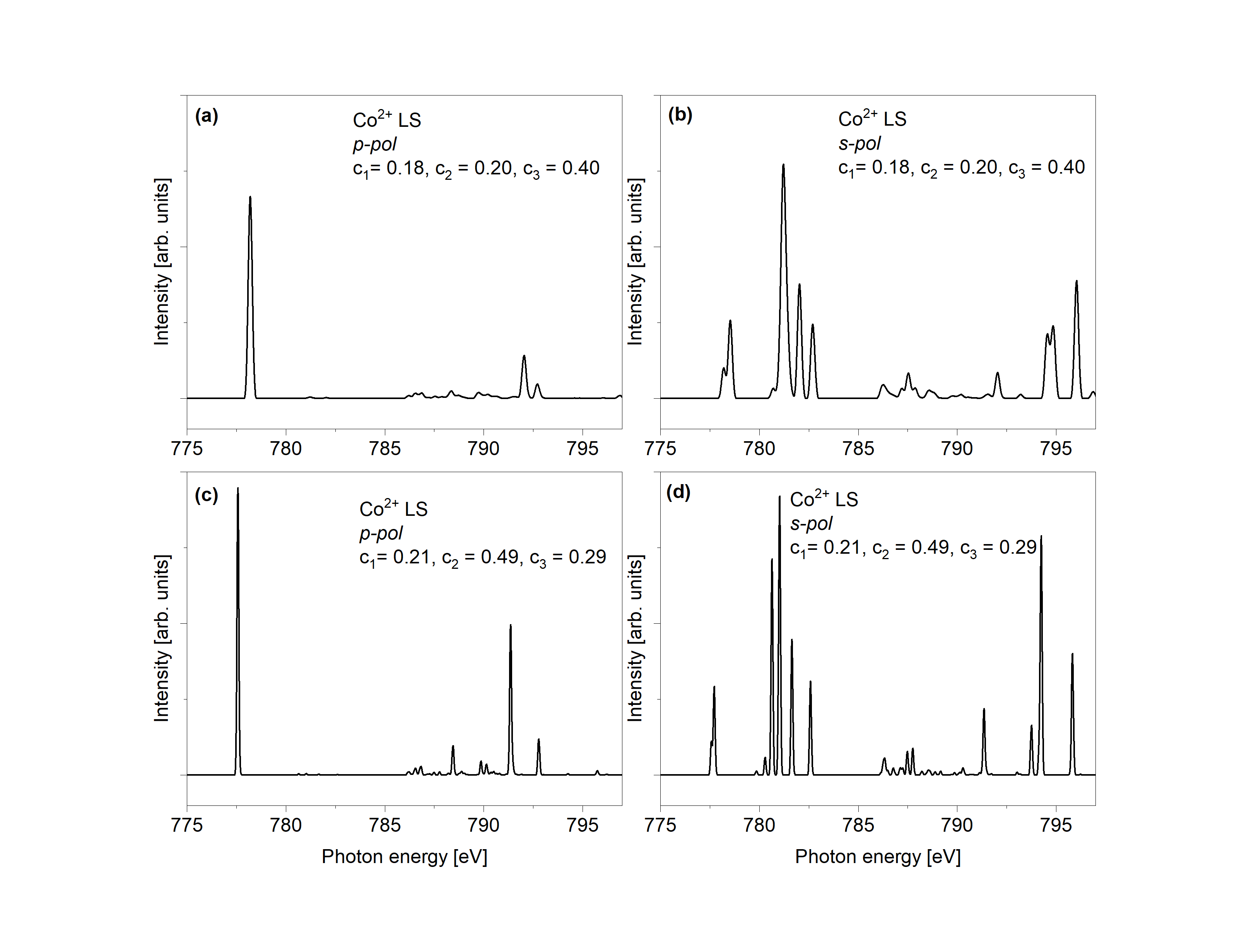


**Figure S7.** Comparison of Co^2+^ LS L_3,2_-edges XAS spectra calculated using two different sets of semi-empirical ROCIS parameters. Spectra obtained with (c_1_ = 0.18, c_2_ = 0.20, c_3_ = 0.40) are shown for (a) p-polarization and (b) s-polarization, while those calculated with (c_1_ = 0.21, c_2_ = 0.49, c_3_ = 0.29) are shown for (c) p-polarization and (d) s-polarization. All simulated spectra were shifted by +16.91 eV and convoluted with a Gaussian broadening of 0.1 eV.

**Influence of functionals on the simulated Co L_3,2_-edges spectra**

Further simulations were carried out using the parameter set c_1_ = 0.21, c_2_ = 0.49, c_3_ = 0.29, as it provided the most consistent spectral features across all tested conditions. Although only minor variations were observed among the different parameter sets, this choice ensures internal consistency and reliable comparison with experimental data (Figures S8 and S9).


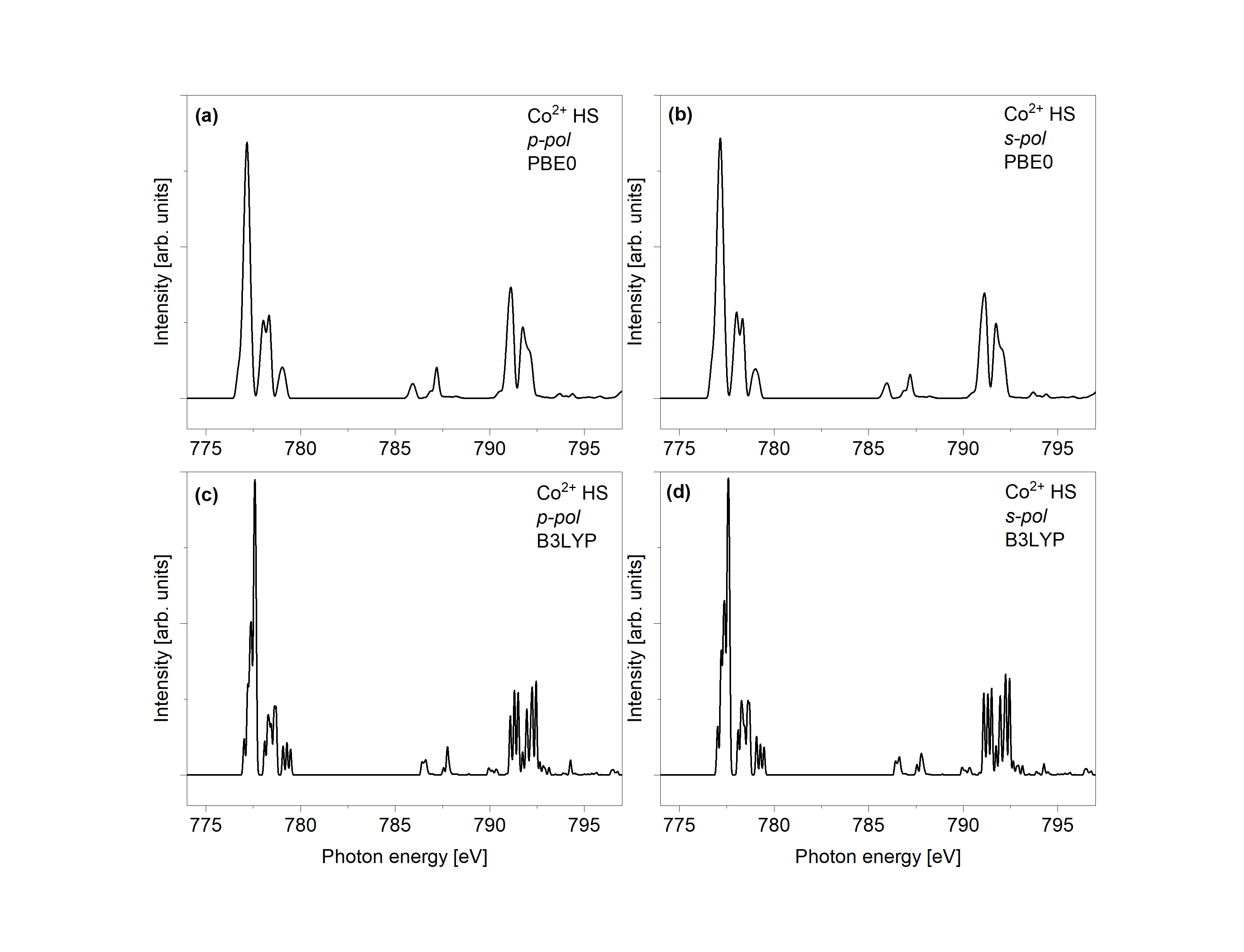


**Figure S8.** Comparison of Co^2+^ HS L_3,2_-edges XAS spectra calculated using two different hybrid functionals. Spectra obtained with PBE0 are shown for (a) p-polarization and (b) s-polarization, while those calculated with B3LYP are shown for (c) p-polarization and (d) s-polarization. B3LYP simulated spectra were shifted by +16.91 eV, while PBE0 by +13.74 eV. All spectra were convoluted with a Gaussian broadening of 0.1 eV.

The simulated Co L_3,2_-edges spectra exhibit a much stronger sensitivity to the choice of exchange–correlation functional than to variations in the semi-empirical ROCIS parameters. For the Co^2+^ LS complex (Figure S9), two main differences are observed. First, the relative intensity distribution between the L_2_ and L_3_ regions varies notably: the PBE0 functional yields lower L_2_ intensities than B3LYP for both polarizations. Second, in the L_3_ region, the B3LYP functional produces a greater number of spectral features with higher intensities, especially under s-polarization. Overall, the B3LYP functional provides a more detailed spectral profile, appearing to better reproduce the experimental complexity of the Co L_3,2_-edges region.


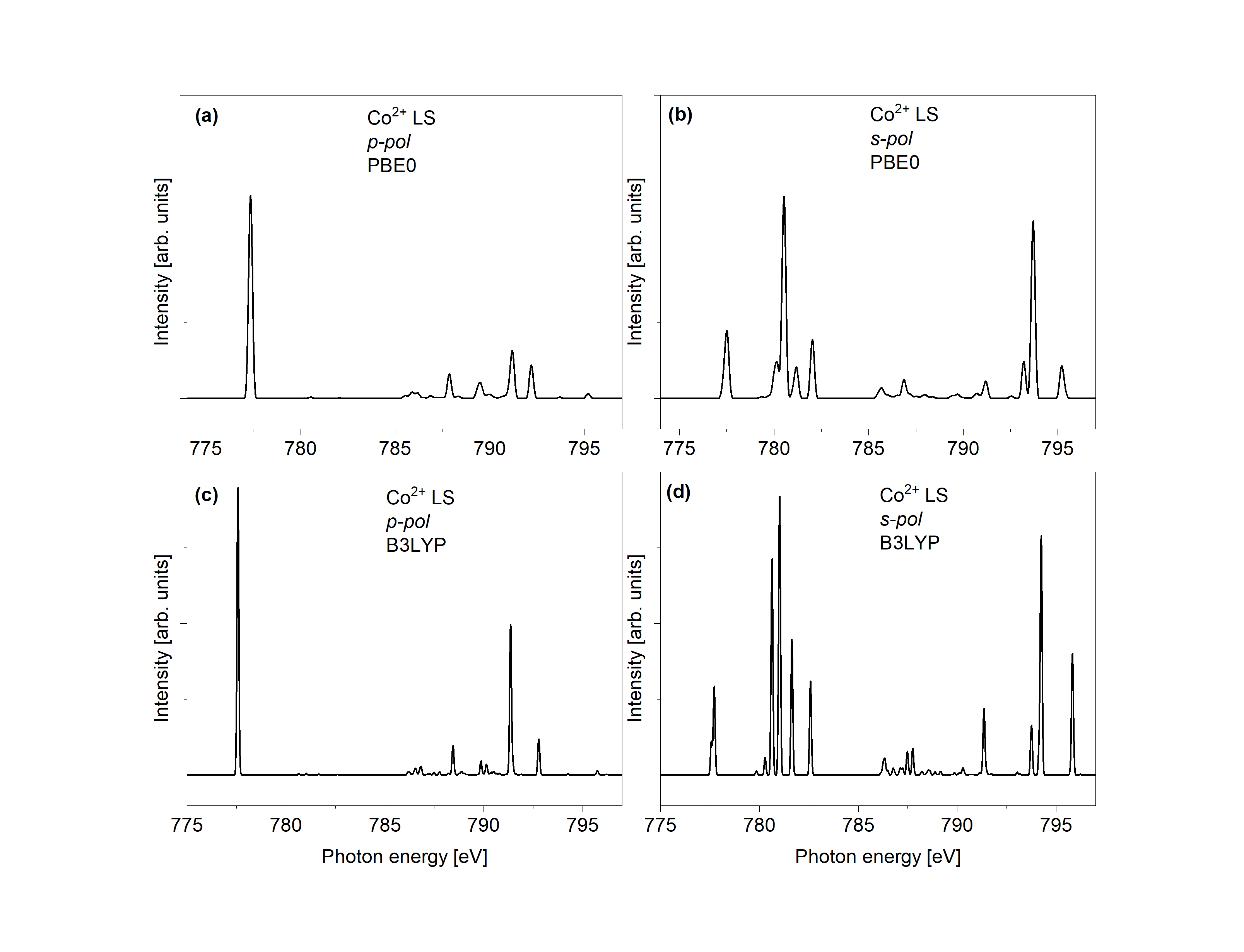


**Figure S9.** Comparison of Co^2+^ LS L_3,2_-edges XAS spectra calculated using two different hybrid functionals. Spectra obtained with PBE0 are shown for (a) p-polarization and (b) s-polarization, while those calculated with B3LYP are shown for (c) p-polarization and (d) s-polarization. B3LYP simulated spectra were shifted by +16.91 eV, while PBE0 by +13.74 eV. All spectra were convoluted with a Gaussian broadening of 0.1 eV.

**S7. Optimized coordinated for HS and LS states and ORCA input files for L_3,2_-edges simulated XAS spectra**

**Table S1.** Optimized coordinated for Co^2+^ LS

Co -1.701294 0.014113 10.941894

C 1.248856 4.443584 10.870383

C 1.457098 3.116550 10.490882

C 0.460548 2.145644 10.677869

C 2.645816 2.662304 9.860949

N -0.359350 1.344253 10.799956

C -4.662081 -4.409808 10.996786

C -4.866750 -3.083318 11.380058

C -6.056011 -2.626763 12.007307

C -3.866235 -2.115218 11.199748

N -3.043367 -1.315916 11.084095

C -4.589134 4.482847 10.983859

C -4.815531 3.162982 11.377753

C -6.006849 2.733847 12.020243

C -3.834852 2.175307 11.194753

N -3.027501 1.360685 11.076644

N -0.375275 -1.332834 10.806048

C 1.175582 -4.460470 10.880409

C 1.405168 -3.140107 10.490005

C 0.428705 -2.149686 10.680620

C 2.595070 -2.713170 9.843466

H -6.199984 -3.161264 12.945124

H -6.905686 -2.809591 11.351146

H -5.974448 -1.559286 12.206710

H 2.712309 -3.253462 8.905244

H 3.450111 -2.913976 10.487285

H 2.535383 -1.644632 9.642007

H -3.727694 -4.700543 10.518961

H -5.436789 -5.153970 11.175591

H 1.944474 -5.215803 10.724852

H 0.227577 -4.735221 11.340540

H -3.654350 4.752311 10.494483

H -5.347292 5.243087 11.165935

H 2.033488 5.183135 10.717621

H 0.301759 4.739342 11.319221

H -6.859620 2.929224 11.371760

H -6.130468 3.276951 12.956020

H -5.944130 1.666245 12.225703

H 3.497127 2.850526 10.513455

H 2.784458 3.194149 8.920821

H 2.567537 1.593859 9.665452

**Table S2.** Optimized coordinated for Co^2+^ HS

Co 0.655264 0.268928 0.265454

N 0.774717 0.255715 2.238679

N 2.449900 0.230282 -0.563022

N -0.485651 -1.306129 -0.091123

N -0.134703 1.897721 -0.529531

C -0.519830 2.823001 -1.103684

C -0.980958 3.933648 -1.826467

C -2.390562 4.061515 -1.936530

C -0.080027 4.818225 -2.423441

C -1.217464 -2.194815 -0.185115

C -2.126237 -3.259273 -0.282334

C -2.893153 -3.313871 -1.475811

C -2.272816 -4.168871 0.767257

C 0.822600 0.103946 3.382635

C 0.887744 -0.112632 4.767433

C -0.069435 -0.902572 5.407861

C 1.989310 0.478860 5.439689

C 3.485825 0.343228 -1.061085

C 4.745958 0.510637 -1.655068

C 5.826039 -0.158074 -1.021355

C 4.898959 1.325496 -2.778837

H 1.959833 1.558992 5.304164

H 2.918444 0.086682 5.028803

H 1.934688 0.244653 6.501802

H 5.635128 -1.230185 -1.014111

H 5.924251 0.200073 0.002366

H 6.747271 0.042919 -1.566198

H -2.226358 -3.405710 -2.331889

H -3.482339 -2.402948 -1.570682

H -3.559038 -4.174835 -1.440337

H -2.826200 4.145379 -0.941991

H -2.796591 3.184184 -2.437880

H -2.630179 4.953067 -2.514147

H -2.981478 -4.991150 0.680206

H -1.678450 -4.055852 1.672727

H -0.010502 -1.064899 6.483081

H -0.874757 -1.356225 4.832034

H -0.449294 5.676988 -2.982090

H 0.991735 4.649205 -2.330278

H 5.881330 1.448789 -3.232342

H 4.035897 1.837629 -3.201644

**Table S3.** ORCA input file for Co²⁺ LS L_3,2_-edges XAS spectra

%basis

AuxC "def2/J"

end

!def2-TZVP def2-TZVP/C B3LYP D3BJ TightSCF DEFGRID3

%rocis NRoots 125

MaxDim 250

SOC true

DoRI true

PrintLevel 3

DoLowerMult true

DoHigherMult true

OrbWin = 2,4,0,2000

DoDFTCIS true

DFTCIS_C = 0.21, 0.49, 0.29

end

* xyz 2 2

Co -1.701294 0.014113 10.941894

C 1.248856 4.443584 10.870383

C 1.457098 3.116550 10.490882

C 0.460548 2.145644 10.677869

C 2.645816 2.662304 9.860949

N -0.359350 1.344253 10.799956

C -4.662081 -4.409808 10.996786

C -4.866750 -3.083318 11.380058

C -6.056011 -2.626763 12.007307

C -3.866235 -2.115218 11.199748

N -3.043367 -1.315916 11.084095

C -4.589134 4.482847 10.983859

C -4.815531 3.162982 11.377753

C -6.006849 2.733847 12.020243

C -3.834852 2.175307 11.194753

N -3.027501 1.360685 11.076644

N -0.375275 -1.332834 10.806048

C 1.175582 -4.460470 10.880409

C 1.405168 -3.140107 10.490005

C 0.428705 -2.149686 10.680620

C 2.595070 -2.713170 9.843466

H -6.199984 -3.161264 12.945124

H -6.905686 -2.809591 11.351146

H -5.974448 -1.559286 12.206710

H 2.712309 -3.253462 8.905244

H 3.450111 -2.913976 10.487285

H 2.535383 -1.644632 9.642007

H -3.727694 -4.700543 10.518961

H -5.436789 -5.153970 11.175591

H 1.944474 -5.215803 10.724852

H 0.227577 -4.735221 11.340540

H -3.654350 4.752311 10.494483

H -5.347292 5.243087 11.165935

H 2.033488 5.183135 10.717621

H 0.301759 4.739342 11.319221

H -6.859620 2.929224 11.371760

H -6.130468 3.276951 12.956020

H -5.944130 1.666245 12.225703

H 3.497127 2.850526 10.513455

H 2.784458 3.194149 8.920821

H 2.567537 1.593859 9.665452

*

**Table S4.** ORCA input file for Co^2+^ HS L_3,2_-edges XAS spectra

%basis

AuxC "def2/J"

end

!def2-TZVP def2-TZVP/C B3LYP D3BJ TightSCF DEFGRID3

%rocis NRoots 125

MaxDim 250

SOC true

DoRI true

PrintLevel 3

DoLowerMult true

DoHigherMult true

OrbWin = 2,4,0,2000

DoDFTCIS true

DFTCIS_C = 0.21, 0.49, 0.29

end

* xyz 2 4

Co 0.655264 0.268928 0.265454

N 0.774717 0.255715 2.238679

N 2.449900 0.230282 -0.563022

N -0.485651 -1.306129 -0.091123

N -0.134703 1.897721 -0.529531

C -0.519830 2.823001 -1.103684

C -0.980958 3.933648 -1.826467

C -2.390562 4.061515 -1.936530

C -0.080027 4.818225 -2.423441

C -1.217464 -2.194815 -0.185115

C -2.126237 -3.259273 -0.282334

C -2.893153 -3.313871 -1.475811

C -2.272816 -4.168871 0.767257

C 0.822600 0.103946 3.382635

C 0.887744 -0.112632 4.767433

C -0.069435 -0.902572 5.407861

C 1.989310 0.478860 5.439689

C 3.485825 0.343228 -1.061085

C 4.745958 0.510637 -1.655068

C 5.826039 -0.158074 -1.021355

C 4.898959 1.325496 -2.778837

H 1.959833 1.558992 5.304164

H 2.918444 0.086682 5.028803

H 1.934688 0.244653 6.501802

H 5.635128 -1.230185 -1.014111

H 5.924251 0.200073 0.002366

H 6.747271 0.042919 -1.566198

H -2.226358 -3.405710 -2.331889

H -3.482339 -2.402948 -1.570682

H -3.559038 -4.174835 -1.440337

H -2.826200 4.145379 -0.941991

H -2.796591 3.184184 -2.437880

H -2.630179 4.953067 -2.514147

H -2.981478 -4.991150 0.680206

H -1.678450 -4.055852 1.672727

H -0.010502 -1.064899 6.483081

H -0.874757 -1.356225 4.832034

H -0.449294 5.676988 -2.982090

H 0.991735 4.649205 -2.330278

H 5.881330 1.448789 -3.232342

H 4.035897 1.837629 -3.201644

*

**REFERENCES**

[64] B. T. Thole, P. Carra, F. Sette, G. Van Der Laan, “X-Ray Circular Dichroism as a Probe of Orbital Magnetization” *Phys Rev Lett* **1992**, *68*, 1943–1946.

[65] P. Carra, B. T. Thole, M. Altarelli, X. Wang, “X-Ray Circular Dichroism and Local Magnetic Fields” *Phys Rev Lett* **1993**, *70*, 694.
